# Reconstruction of Upper Eyelid Defects Secondary to Malignant Tumors with a Newly Modified Cutler-Beard Technique with Tarsoconjunctival Graft

**DOI:** 10.1155/2019/6838415

**Published:** 2019-03-03

**Authors:** Álvaro Bengoa-González, Bianca Ma Laslău, Agustín Martín-Clavijo, Enrique Mencía-Gutiérrez, Ma Dolores Lago-Llinás

**Affiliations:** ^1^Ophthalmology Department, 12 de Octubre Hospital, Complutense University, 28041 Madrid, Spain; ^2^Dermatology Department, Queen Elizabeth Hospital Birmingham, B15 2TH Birmingham, UK

## Abstract

**Purpose:**

We describe a modification of Cutler-Beard's technique, using a contralateral tarsoconjunctival graft, in patients who underwent excision of large malignant tumors of the upper eyelid.

**Methods:**

This is an interventional retrospective review (clinical study) of ten cases, with age range from 64 to 86 years (mean: 71.2 years ± 6.49) with malignant upper eyelid tumors, operated by the same surgeon (AB-G), between 2011 and 2016. The resulting defects were reconstructed using a modification of Cutler-Beard's technique. A tarsoconjunctival graft was harvested from the contralateral upper eyelid, with an extra 3 mm of conjunctiva from the superior edge of tarsus and was attached to the upper eyelid defect, different from that previously described. Follow-up ranged between 24 and 60 months (mean: 41.6 ± 9.87). Functional, cosmetic outcomes and postoperative complications were evaluated.

**Results:**

No upper eyelid retraction, eyelid margin entropion, or graft retraction was observed in any of the cases. All of the patients were satisfied with the aesthetic result.

**Conclusion:**

This technique allows us to safely inset a suitable graft on the ocular surface, with sufficient blood supply, resulting in a stable margin and good contour.

## 1. Introduction

In the reconstruction of large full-thickness upper eyelid defects, the technique described by Cutler and Beard in 1955 is still commonly used [1]. Many modifications of this technique have been described over the years, with the aim of improving outcomes.

The reconstruction of upper eyelid defects is a major challenge as it has a number of considerations and difficulties not found in the reconstruction of lower eyelid defects and the techniques used are under constant review. The primary role of the upper eyelid is to protect the ocular surface, but it also has a dynamic function; it requires a mucosal inner surface and a stable margin in order to maintain an adequate mobility and avoid abrasion of the ocular surface that could endanger visual quality [2–4].

In addition, we cannot forget that cosmesis is a very important factor for the patient, and poor eyelid reconstruction can lead to psychosocial morbidity; therefore, for a successful reconstruction, we need to maintain a good contour and appearance in addition to a good functional outcome.

When the defects are less than one-third of the eyelid, they can be closed directly if lid laxity allows it, but when they are bigger than 50%, then flaps and grafts will need to be utilized [5].

The classic Cutler-Beard technique (CBT) is a procedure that is performed in two stages. Firstly, a full-thickness flap (conjunctiva-muscle-skin) from the ipsilateral lower eyelid is created, released from the lower edge of the tarsus, advanced under the bridge formed by the tarsus and the lid margin, and inset to the upper eyelid defect. After 6–8 weeks, the flap is divided and the pedicle is returned to its original position in the lower eyelid.

Upper eyelid retraction and entropion are frequent complications of this procedure due to the absence of rigid posterior lamellar support. Several modifications have been used to address these issues. Autologous tissues are more resistant to resorption, with the tarsoconjunctival grafts, and the most suitable for the reconstruction of the posterior lamella because of its conjunctival posterior surface, its simple technique and low donor site morbidity [6, 7].

In this paper, we describe a new modification of the CBT, using a contralateral tarsoconjunctival graft attached to the upper eyelid defect different from that previously described by other authors. With this technique, we achieved a suitable graft placement overlying the ocular surface, adequate blood supply and, in the second step, a stable lid margin and a similar contour to the original state.

## 2. Methods

From 2011 through 2016, we recruited 10 patients (5 male and 5 female, age range from 64 to 86 years) with malignant tumors of the upper eyelid that required excision of at least 60% of the eyelid. Four tumors were squamous cell carcinomas, two sebaceous cell carcinomas, three infiltrative basal cell carcinomas, and one Merkel cell carcinoma (Table 1). All cases were reconstructed with the newly modified technique, using contralateral tarsoconjunctival graft. The follow-up ranged from 24 to 60 months (mean: 41.6 ± 9.87 months).

This clinical study was approved by the Ethics Committee of the 12 de Octubre Hospital (approval number 18/368), Complutense University of Madrid, Spain. The study was registered at Clinicaltrials.gov with the identifier: NCT03712995.

### 2.1. Surgical Technique

**Figure 1:**
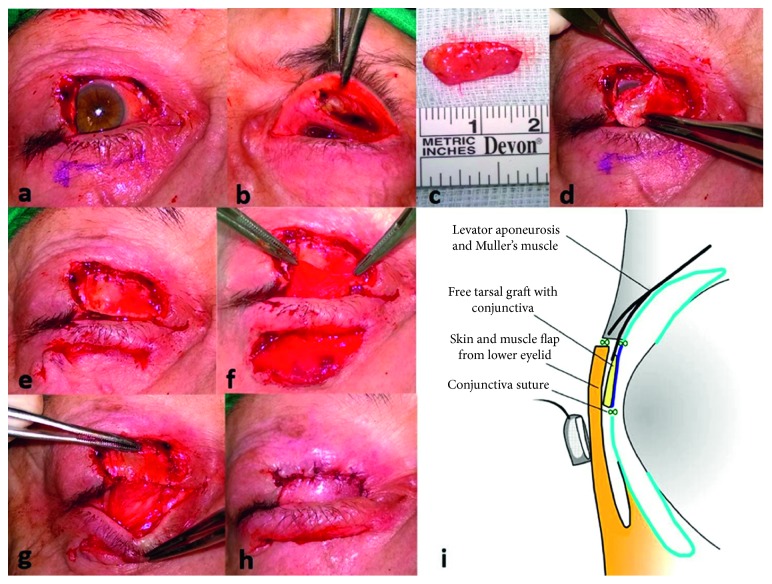


**Figure 2:**
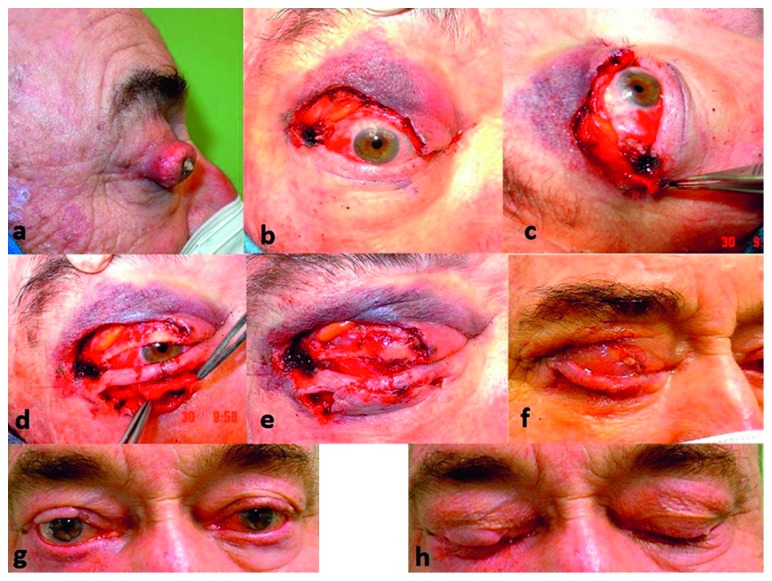


All cases were treated with surgical excision with margin control. In each stage, a 3 mm margin was taken, with further stages taking every 48–72 hours until histological clearance was achieved (Figures 1(a), 2(a), and 2(b)). The reconstruction was carried out 48 hours later.

In the first reconstructive stage, the contralateral tarsoconjunctival graft was harvested using a No. 15 blade, with a perpendicular incision, starting 4 mm from the lid margin and including 3 mm of conjunctiva from the superior edge of the tarsus, dissecting it from the aponeurosis and Muller's muscle (Figure 1(b)). The graft length, depending on the size of the defect to be reconstructed, may reach up to 17 mm (Figure 1(c)). The donor site was allowed to heal by secondary intention. The graft was placed into the upper eyelid defect, aligning the lower tarsal edge with the edges of the defect, with the conjunctival side facing the ocular surface. The excess conjunctival side from the superior edge of the graft was sutured superiorly to the conjunctiva or to the superior retractor muscles and laterally and medially to the margins of the defect or to the canthal tendon (Figures 1(d) and 1(e)). This allowed a complete reconstruction of the posterior lamella.

The size of the lower eyelid flap was designed to cover the surgical defect. With a No. 15 scalpel, a full thickness incision 1-2 mm below the lower tarsal edge was done to create a bridge between the lid margin and the tarsus. This was followed by two vertical incisions, down to but not including posterior lamella to release the flap. We then separated the posterior lamella of the flap (formed by conjunctiva) from the anterior lamella (formed by skin and orbicularis muscle) (Figure 1(f)). Once separated, both lamellas were mobilized under the bridge. The conjunctiva was sutured with 7/0 absorbable polyglactin 910 to the lower edge of the tarsoconjunctival graft (Figures 1(g) and 2(e)), and the skin and muscle of the flap were attached to the upper skin defect over the graft, with 6/0 polypropylene sutures (Figures 1(h) and 2(f)).

The second stage, the separation of the flap was carried out 3 weeks later at the level of the defect margins. Once the flap pedicle was divided, the anterior lamella was sutured (using absorbable 7/0 sutures) to the tarsoconjunctival graft in order to reconstruct the margin. The remaining conjunctiva of the lower flap was taken back to its original position and along with the lower eyelid retractors was sutured to the lower tarsal edge with absorbable 6/0 sutures. The same was done with the excess skin and muscle of the flap, restoring the anatomy of the lower eyelid.

One patient had a large defect, which passed the lateral canthus, so the reconstruction was extended with a periosteal flap from the lateral orbital rim, to allow suturing the tarsoconjunctival graft (Figures 2(c) and 2(d)).

All patients were followed-up 1, 3, 6, and 12 months after final reconstruction, followed by annual review. At the time of writing this paper, 8 patients showed no signs of recurrence; one patient died from unrelated causes and another from systemic metastases.

## 3. Results

**Figure 3:**
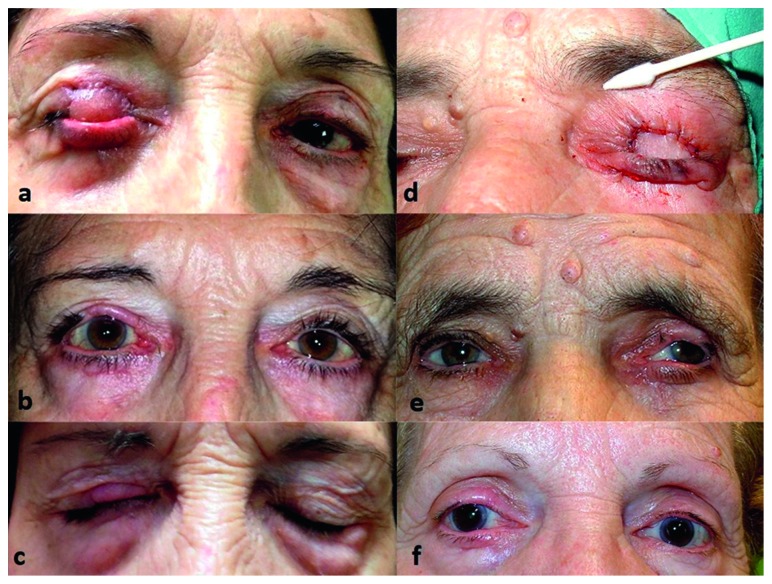


The only postoperative complication was mild ocular discomfort when blinking during the 3 days following the separation of the flap which settled with the use of ocular lubricants; no hyperemia or ocular surface disorders were observed. No graft necrosis or flap dehiscence was noted (Figures 3(a) and 3(d)).

Long-term complications were minimal. In half of the cases, we observed minimal retraction of the upper lid, never greater than 1 mm, after 12 months, with all patients perfectly able to occlude the eyelids; no cases of lagophthalmos were observed (Figures 2(h) and 3(c)). There was no palpebral retraction on the graft donor site and no retraction or ectropion of the flap donor lower eyelid (Table 2). All patients have good margin stability, with no entropion after 12 months (Figures 2(g), 3(b), 3(e), and 3(f)). All patients are satisfied with the aesthetic result. There were no donor site complications such as discomfort, bleeding, retraction, or entropion.

## 4. Discussion

Upper eyelid large defect reconstructions can be necessary following cancer surgery or other causes such as trauma. The aim of the eyelid reconstruction surgery is to get good eyelid mobility, perfect corneal protection, good aesthetic result, and acceptable sequelae of the donor site. The CBT has proved to be a suitable way of meeting these goals because the flap provides a perfect integration into the recipient bed [5]. Over the years, changes have been made to this technique to further improve the results.

Cutler and Beard described reconstructing upper eyelid defects using a conjunctival-myocutaneous flap from the lower eyelid, mentioning lower eyelid retraction as the only notable complication. Carroll [8] observed that the main problems of this technique is the lack of rigid support in the upper eyelid posterior lamella, causing entropion and ocular surface abrasions that can lead to eye irritation. Other authors [9–11] have performed a lower tarsoconjunctival flap that would advance over the defect of the upper eyelid, sometimes requiring at least 3 mm of superior tarsus to achieve the desired stability [10]. Smith and Obear [12] described a modification that included part of the lower tarsus distal margin in the advancement flap on the upper eyelid to increase its stability.

When large tumor resections are required, the upper lid defect can be left with minimal or no tarsus left. If, the eyelid does not have enough laxity, shortening of the posterior lamella can happen when performing a tarsoconjunctival flap, contributing even more to lower eyelid instability. The risk of destabilizing the donor eyelid is reduced if we can preserve the entire lower eyelid tarsus.

Twenty-five years after the original CBT, entropion due to retraction of the posterior lamella was described. This led Wesley and McCord [13] to use allogeneic sclera as a spacer between the conjunctiva and orbicularis. Carroll [8] suggested that sclera may also undergo significant retraction and recommended autologous auricular cartilage graft as an alternative. Hard palate mucoperiosteum provides a suitable substitute for the posterior lamella, as it contains similar fibrous and mucosal elements to tarsus [14]. The hard palate can provide enough tissue to reconstruct the entire upper eyelid, and donor site complications are generally limited to bleeding; complications in the recipient upper eyelid include ocular irritation, transient keratopathy, partial graft dehiscence, upper eyelid retraction, and necrosis of the overlying skin flap. Corneal irritation is thought to be caused by patches of keratin present in the stratified squamous epithelium of the hard palate. Contraction of hard palate grafts tends to be mild and can be compensated for by modest oversizing of the graft. Nasal septal chondromucosa is another alternative for rigid, mucosalized composite graft material and can be readily harvested from the nasal septum, offering abundant nonkeratinized epithelium that is well-tolerated [15]. Our opinion is that nasal septal chondromucosa may be preferable as an option for lower eyelid rather than upper eyelid reconstruction as the result of its rigidity and less potential for corneal epitheliopathy [16]. Graft materials including auricular cartilage and nasal septum provide rigidity, but do not provide the conjunctival surface that is necessary for contact with the cornea [15]. Other allografts used in this surgery are Achilles tendon [17], irradiated aorta, and allogeneic irradiated tarsus [18], but in small numbers and with similar complications.

Allografts are attractive because they are ready to use in surgery, reduce surgical time and there is no donor site sequelae. However, the disadvantages are obvious: the economic cost, availability, risk of rejection, and a potential risk of disease transmission. Very important is the unpredictable resorption rate, decreasing its effectiveness over time [19]. Contrarily, autologous grafts have the advantage of minimal resorption and no risk of transmitted diseases and graft rejection, but it carries the risk of donor site morbidity.

The tarsoconjunctival grafts are used in many eyelid reconstruction techniques [20]. Leibovitch et al. [6] reported significant retraction compared to palate grafts, but with fewer complications. The cause of this is unclear. In their article, they do not discuss details of placement, positioning, or graft attachment during reconstruction of the upper eyelid posterior lamella. We believe that to prevent graft shrinkage or eyelid retraction, appropriate placement is needed with no graft tension, and a good blood supply while the flap is attached. The lower eyelid myocutaneous flap placed over the graft ensures greater vascularization of the graft in addition to providing muscle fibre to ensure the function of the resulting anterior lamella with adequate eyelid closure.

Rajak et al. [21] have recently described a modification of CBT using a tarsoconjunctival graft. They raise a lower lid cutaneous flap, like Hsuan and Selva [2], over the graft attached to the defect. When the flap is separated at 2–4 weeks, traction is placed on the eyelids for a few days. This led in some cases to dehiscence and necrosis at the junction of the flap and severe pain until division, likely due to corneal abrasion.

In our series, we did not observe flap dehiscence or corneal abrasions. This could be explained by the careful placing and suturing of the graft to the inferior conjunctival flap as well as to the upper conjunctiva without tension, thus maximizing the vascular supply, at the same time as protecting the ocular surface and the cornea.

Like Hsuan and Selva [2], we observed no changes in the upper eyelid height or occlusion. They used contralateral tarsal graft in the first stage of a modified Cutler-Beard advancement flap using only lower eyelid skin. They argue that a skin-only flap allows earlier division. In one patient, they noticed mild lower eyelid ectropion following earlier division.

We believe an earlier separation would not compromise vascularity, but could produce eyelid retraction, as it has been observed in other cases of early separation in Hughes flap reconstructions [22].

Yoon and McCulley [3] reported that a secondary tarsoconjunctival graft placement may avoid the challenge of dividing the eyelid precisely at the border of the tarsal graft, simplifying the procedure. Their modification also allows coverage of the eyelid margin with conjunctiva.

Our procedure can be safely performed at an early stage, by maintaining an adequate blood supply to the graft and also by having enough conjunctiva for the eyelid margin reconstruction once the flap is divided. This division is carried out in the third week, providing enough time in which this flap exerts traction in the superior eyelid over the lower eyelid. To ensure a proper margin reconstruction, we divide the flap, making the cut from the posterior lamella provided some more tissue, which allowed us to suture it to the anterior lamella. Then, the lower excess conjunctiva and the retractors are sutured together to the inferior tarsal edge, as well as the skin and orbicularis muscle, creating lower eyelid anatomical structure restitution, avoiding the need for traction after flap division. Therefore, the conjunctiva does not need to extend to the superior edge of the under eyelid defect. The conjunctiva receives less tension and the risk of lower eyelid malposition due to scarring also decreases.

Leibovitch and Selva and McKelvie et al. reported erythema and hypertrophy of the newly formed eyelid margin following division of the tarsoconjunctival flap in lower eyelid reconstruction [23, 24]. Although this problem is infrequent, our experience suggests that this can be minimized by suturing the cutaneoconjunctival edge with 6/0 polyglactin 950 suture. This observation also had been made by other authors [25, 26]. For this reason, we use the same technique in the upper eyelid reconstruction.

Tarsoconjuntival grafts have their limitations. These are related to the graft size that can be removed without risk of damaging the donor area. The superior tarsus is on average 11 mm in height and 28 to 30 mm in length. It has been reported [3] that it is safe to take up to 17 mm in length and 6-7 mm in height, preserving 4 mm adjacent to the eyelid margin. We believe that this size is enough to reconstruct upper eyelid defects that do not cross the lateral or medial canthus and preserve some tarsal tissue on each side of the defect where the graft will be attached. In defects or those that cross the medial or lateral canthus, orbital rim periosteal flaps can be used to attach the tarsoconjunctival graft, as seen in one of our cases, achieving margin and graft stability [16].

The alternatives in recovery are varied [27], although the reconstruction of the upper eyelid must respect mobility, flexibility, function, and sufficient conjunctival surface to protect the cornea [28]. Inaccurate concepts in lamellar grafts can lead to failures. Alternatives such as nasal septal and ear cartilage donor tissue can produce aesthetic and functionally satisfactory results. However, the risk of donor site complications is high [29].

We consider that tarsoconjunctival grafts are the ideal tissue for both lower and upper eyelid posterior lamella defects with excellent functional and aesthetic results. It minimally extends the surgical time and has few or no complications at the donor site [7].

In conclusion, this technique allows us to completely reconstruct the upper eyelid defect with the required rigidity, support, and full eyelid mobilization without irritating the ocular surface. The union of the graft by the conjunctival flap to the upper retractors and its attachment to the lower conjunctiva reduces traction and the possibility of dehiscence of the myocutaneous flap [18]. In addition, it achieves good lid margin contour, with appearance and stability similar to the contralateral eyelid (Figures 2(g), 3(b), 3(e), and 3(f)). Despite being a technique that occludes the vision for 3 weeks, the end result achieved ensures full functional recovery and very good cosmetic result. However, this technique is limited because it is not adequate in patients with low vision of the contralateral eye, and it is a 2 stage procedure.

More reconstruction cases with this promising technique should be performed in order to consolidate our findings and identify any defects that may have gone unnoticed in this study.

## Figures and Tables

**Table 1:** Patients, tumors characteristics, surgical procedures used in upper eyelid surgery, follow-up, postoperative complications, and final results.

Patient	Gender/age (years)	Upper lid tumor	Defect size (mm)	Upper lid surgery	Preoperative size graft length × height (mm)	Follow-up (months)	Postoperative complications	Final outcome
1	F/86	MCC right	24 × 12	CBT + FTG	17 × 5	24	Ocular discomfort topical treatment few days	Good final contour and margin. Minimal retraction.
2	M/66	SCC right	17 × 10	CBT + FTG	14 × 5	48	None	Good final contour and margin. No retraction.
3	F/70	BCC left	21 × 10	CBT + FTG	16 × 5	48	None	Good final contour and margin. Minimal retraction.
4	M/72	SCC left	20 × 12	CBT + FTG	15 × 5	38	None	Good final contour and margin. No retraction.
5	F/68	SCC left	22 × 10	CBT + FTG	17 × 5	36	None	Good final contour and margin. Minimal retraction.
6	F/64	SC right	18 × 12	CBT + FTG	14 × 5	42	Ocular discomfort topical treatment few days	Good final contour and margin. Minimal retraction.
7	F/72	BCC left	20 × 10	CBT + FTG	15 × 5	36	None	Good final contour and margin. No retraction
8	M/67	SCC right	24 × 10	CBT + FTG + PF	17 × 5	36	None	Good final contour and margin. No retraction.
9	M/78	BCC left	18 × 10	CBT + FTG	14 × 5	48	None	Good final contour and margin. Minimal retraction
10	M/69	SC left	18 × 12	CBT + FTG	14 × 5	60	Ocular discomfort topical treatment few days	Good final contour and margin. No retraction.

M = male, F = female, mm = millimeters, MCC = Merkel cell carcinoma, BCC = basal cell carcinoma, SCC = squamous cell carcinoma, SC = sebaceous carcinoma, CBT = Cutler-Beard Technique, FTG = free tarsoconjunctival graft, PF = periosteal flap.

**Table 2:** Postoperative upper lid MRD and preoperative and postoperative lower lid MRD of the operated eyelid at 12 months.

Patient	Postoperative upper lid MRD of the right and left eyes at 12 months (mm)	Preoperative and postoperative lower lid MRD of the eyelid operated at 12 months (mm)
1	R3 (surgical site) L4	R6 (preop) R6 (postop)
2	R4 (surgical site) L4	R5 R5
3	R4 L3 (surgical site)	L6 L6
4	R3L3 (surgical site)	L6 L6
5	R4L3 (surgical site)	L5 L5
6	R4 (surgical site) L4	R5 R5
7	R3L3 (surgical site)	L6 L6
8	R3 (surgical site) L4	R6 R6
9	R4L3 (surgical site)	R5 R5
10	R3L3 (surgical site)	L6 L6

R = right eye; L = left eye; MRD = margin reflex distance; mm = millimeters.

## Data Availability

The data used to support the findings of this study are available from the corresponding author upon request.
